# Dipolar Noise in Fluorinated Molecular Wires

**DOI:** 10.3390/nano12081371

**Published:** 2022-04-16

**Authors:** Mingyu Jung, Shashank Shekhar, Duckhyung Cho, Myungjae Yang, Jeehye Park, Seunghun Hong

**Affiliations:** Department of Physics and Astronomy, Seoul National University, Seoul 08826, Korea; tririver@snu.ac.kr (M.J.); choduckhyung@gmail.com (D.C.); goodymj@snu.ac.kr (M.Y.); jhpark6521@snu.ac.kr (J.P.)

**Keywords:** self-assembled monolayers, molecular transport, noise, dipole-interaction, tunneling and thermionic conduction

## Abstract

We demonstrate a strategy to directly map and quantify the effects of dipole formation on electrical transports and noises in the self-assembled monolayers (SAMs) of molecular wires. In this method, the SAM patterns of fluorinated molecules with dipole moments were prepared on conducting substrates, and a conducting probe in contact-mode atomic force microscopy was utilized to map currents and noises through the probe on the molecular patterns. The maps were analyzed to extract the characteristic parameters of dipolar noises in SAMs, and the results were compared with those of hydrogenated molecular patterns without dipole moments. At rather low bias conditions, the fluorinated molecular junctions exhibited a tunneling conduction and a resistance value comparable to that of the hydrogenated molecules with a six-times-longer length, which was attributed to stronger dipoles formation in fluorinated molecules. Interestingly, conductance (*G*) in different regions of fluorinated molecular patterns exhibited a strong correlation with a noise power spectral density of *S*_I_/*I*^2^ like *S*_I_/*I*^2^ ∝ *G*^−2^, which can be explained by enhanced barrier fluctuations produced by the dipoles of fluorinated molecules. Furthermore, we observed that the noise power spectral density of fluorinated molecules showed an anomalous frequency (*f*) dependence like *S*_I_/*I*^2^ ∝ 1/*f*^1.7^, possibly due to the slowing down of the tunneling of carriers from increased barrier fluctuations. In rather high bias conditions, conductions in both hydrogenated and fluorinated molecules showed a transition from tunneling to thermionic charge transports. Our results provide important insights into the effects of dipoles on mesoscopic transport and resistance-fluctuation in molecules and could have a significant impact on the fundamental understanding and applications in this area.

## 1. Introduction

A molecular wire (a molecule connected with two metallic reservoirs) [1,2] has been studied extensively as one of the key components for next-generation ultra-high-density electronic devices due to their small sizes, and their high packing densities (~10^14^ cm^−2^) in a thin film structure [3,4,5,6,7,8,9]. For example, a data storage of 41 Tb-cm^−2^ was achieved recently in a molecular monolayer by controlling the dipoles of an individual molecule in ambient conditions [3]. In common cases, molecular wires on solid substrates formed well-ordered self-assembled monolayers (SAMs) and were utilized for surface modification of solid electrodes in various optoelectronic devices such as thin film transistors, light-emitting diodes, solar cells, and memory cells [3,4,5,6,7,8,9,10,11,12,13]. For example, previous works showed that SAM coatings on indium tin oxide (ITO) electrodes in organic solar cells enhanced charge injections into organic materials, resulting in increased solar cell efficiency [9,10,11,12,13].

On the other hand, a molecular wire in a self-assembled monolayer structure is considered as an ideal model system for a fundamental study of one-dimensional transport phenomena. Relatively simple electronic and chemical structures of a molecular wire enable the modification of its transport properties in a controlled manner. A modification in the length or end groups of a molecular wire can induce substantial changes in the electronic properties of SAMs based on molecular wires [14,15,16,17,18,19,20,21,22,23,24,25,26]. For example, fluorinated-SAMs (FSAMs) with a fluorine atom as an end group of the molecular wires have attracted much attention due to their interesting properties such as improved thermal stability, a long lifetime, lubricants, antibiofouling properties, biological and chemical inertness, and high hydrophobicity [18,19,20,21,22,23,24]. The high electronegativity of a fluorine atom results in the formation of a rather strong molecular dipole moment (*μ*) in molecular wires. In this case, the electronic properties of FSAMs can be controlled by changing the degree of fluorination in a terminal or a backbone chain of the molecular wire [18,19,20,21,22,23,24]. Additionally, the strong inter- and intra- molecular dipole interactions of molecular wires may affect the structural and electrical properties of FSAMs [20,24]. For example, a strong dipole moment of a molecular wire could induce some additional defects and disorders in FSAMs based on molecular wires and alter the conductance and electrical noises of molecular wires. However, there are only a few studies on the effect of dipolar interactions on electrical noises, and on conductance, in such fluorinated molecular wires. Recently, we have developed a method to directly map localized charge transports and noise source activities in the self-assembled monolayers of molecular wires, while the effect of localized dipole noise sources in molecular wires has not been explored before [7,14].

Herein, we report observations of dipole noises generated by the fluctuations of dipole moments in fluorinated molecular wires and the mapping of such dipole fluctuations affecting the charge transports in the molecular layer. In this strategy, the SAM of fluorinated molecular wires (FSAM) and that of hydrogenated molecular wires (HSAM) were first coated on a specific region of conducting ITO substrates, and a conducting probe in contact with the SAM was utilized to map the electrical currents and noises from molecular wires. The measured maps on FSAM and HSAM were analyzed and compared to identify the electrical noises generated by the dipole fluctuations in fluorinated molecular wires. The FSAM exhibited asymmetric electrical current and noise behaviors depending on the bias polarities, which were attributed to the strong dipole formations in the fluorinated molecular wires. Furthermore, at a rather low bias condition, the normalized noise power spectral density (*PSD*) measured from different regions of the FSAM was inversely proportional to the square of its conductance on the same regions like *PSD* ∝ *G*^−2^, whereas that of the HSAM did not show any dependence. Such scaling behavior of the FSAM was explained by the tunneling barrier fluctuations that originated from the strong dipole moments on the FSAM surface. Interestingly, the noise *PSD* from the FSAM depended on the noise frequency *f* like *PSD* ∝ 1/*f*^1.7^, unlike common scaling behavior, such as *PSD* ∝ 1/*f*^2^, for molecular wires. This could be explained by the slowing down of the tunneling of carriers in the FSAM originating from strong molecular dipoles. At a rather large bias, the noise *PSD* and junction conductance *G* exhibited a correlation like *PSD* ∝ *G*^2^, which was attributed to the thermionic conduction at the high bias condition. Our results can provide valuable insights about noise generations by dipole fluctuations in molecular wires and enable a new understanding of charge transport phenomena in molecular wire-based devices.

## 2. Materials and Methods

Trimethoxy(3,3,3-trifluoropropyl) silane (TTPS) of purity ≥97% (product id 91877) and octadecylsilane (OTS) of purity ≥90% were purchased from Sigma-Aldrich (Buchs, Switzerland). ITO substrates with a low sheet resistance (~8 Ω-□^−1^) and a smooth surface were also obtained from Sigma-Aldrich (Saint Louis, MO, USA) (Product ID 703192). Polydimethylsiloxane (PDMS) and curing agent of silicone elastomer were purchased from Dow Corning Corporation, Midland, MI, USA. A micro-contact printing method was utilized to pattern OTS or TTPS molecules on an ITO substrate, as reported previously [13,14]. In this process, we first cleaned the ITO substrate via oxygen plasma to minimize impurities and improve the uniformity of the substrate, as reported previously [27]. For the stamping of molecular layer patterns, a PDMS stamp was first dipped in a molecular wire solution with a concentration 10 mM in hexane for one minute. Then, the stamp was dried for 10 s under a gentle flow of N_2_. The inked stamp was directly placed on an ITO substrate for 5 s with a gentle pressure by the hand, so that molecules were transferred from the stamp to the ITO and formed the SAM patterns of desired molecular wires. Finally, the substrate was rinsed with ethanol to remove unbound stray molecular wires, leaving a clean patterned surface. Previous work showed that the Trimethoxy(3,3,3-trifuoropropyl)silane is one of the silanes that formed a stable monolayer without significant aggregation [28]. The unattached molecular silane could be easily removed by rinsing with the solvent. The topography and lateral force images on our molecular patterns show a rather uniform flat surface without any molecular aggregation or multilayer formation (Figure S1, Supplementary Materials), which is consistent with previously-reported results. The prepared molecular layer sample was kept under a low vacuum condition (~µbar) until the electrical measurements were carried out [29].

## 3. Results and Discussion

### 3.1. Mapping of Electrical Currents and Dipolar Noises in Patterned Self-Assembled Monolayer

**Figure 1:**
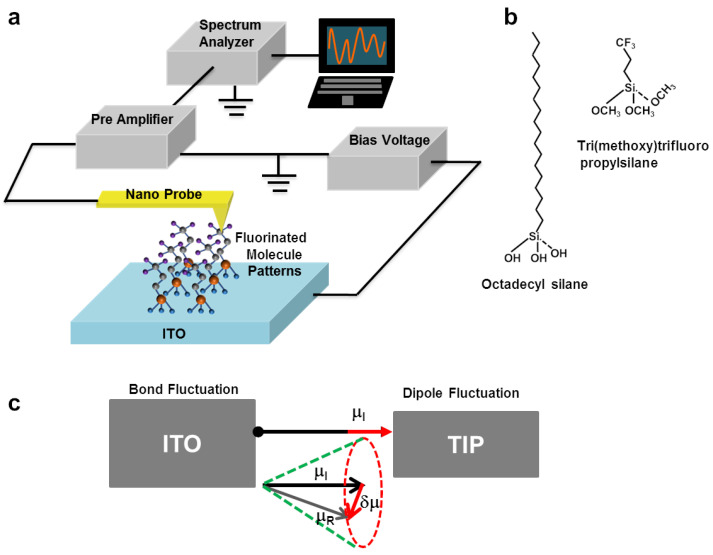
Schematic diagrams showing the scanning noise measurement setup and chemical structures of molecular wires. (**a**) Schematic diagram depicting noise and current measurement setup for a molecular wire pattern. A dc bias was applied to the ITO substrate, and currents and noise signals through the probe were measured by a homemade network analyzer. (**b**) Chemical structures of octadecyl(trihydroxy)silane and tri(methoxy)trifluoropropylsilane molecular wires, which were used to pattern hydrogenated-SAM and fluorinated-SAM, respectively. (**c**) Schematic diagram showing the possible formation of a dipole at the tip. δ*μ* is the change in a dipole moment due to a change in a molecular confirmation or change in position of contact atoms.

Figure 1a shows a schematic illustration of the experimental setup for mapping dipole noises of a molecular wire on the ITO substrate. For noise measurements, a Pt-based conducting probe (25Pt300B, Park System, Suwon-si, Korea) installed on a conducting AFM (XE-70 model of Park System) made direct contact with a molecular wire sample and was used to measure electrical currents through the molecular wires from the ITO substrate. All measurements were performed under ambient conditions at a room temperature of ~24 °C. The contact force on the conducting probe was maintained at 1.0 μN through a feedback loop in the AFM system. It should be mentioned that the diameter of our conducting probe was ~25 nm, implying ~100 times larger tip-substrate contact area than that of the common AFM probe. Such a rather large contact area reduced the pressure by the probe, minimizing the tip-induced damage on the substrate. Furthermore, it allows us to achieve a rather stable tip-substrate junction for reliable conducting AFM mapping [25,26]. During the mapping process, a bias voltage of 0.1 V was applied to the ITO substrate using a function generator (DS345, Stanford Research Systems, Sunnyvale, CA, USA). The measured current was transformed to the amplified voltage signal by a low-noise preamplifier (SR570, Stanford Research Systems). The amplified signals were filtered by a band-pass filter (6 dB) to obtain the absolute electrical noise signal (*S*_I_), which is the fluctuating component of the current signals. The RMS power of the noise signal could be obtained using a home-made RMS-to-DC converter based on an AD737 chip (purchased from Analog Devices, Wilmington, MA, USA). Note that the obtained noise power was the integrated value of the noise *PSD* over the pass band of the used band-pass filter. Finally, we obtained the noise *PSD* value at the central frequency of the pass band by dividing the square of the measured RMS noise power with the bandwidth of the band-pass filter. By scanning the AFM probe on the patterned surface, we could simultaneously obtain the topography, current, and noise *PSD* maps.

### 3.2. Origin of Dipolar Noises in Molecular Wires

Figure 1b shows chemical structures of octadecylsilane (OTS), and Trimethoxy(3,3,3-trifluoropropyl) silane (TTPS) molecules. OTS molecules have eighteen –CH_2_ units in a backbone chain, whereas TTPS molecules have only three –CH_2_ units in the backbone chain. The terminal group of OTS is –CH_3_, whereas TTPS has an electron-withdrawing terminal group of –CF_3_. Both molecular wires can form ordered SAM structures [18,19,20,21,22,23,24].

Figure 1c is the schematic diagram of a molecular wire junction between an ITO substrate and a conducting probe. The heads (tripod atoms) of a molecular wire could make chemi-adsorbed bonds at surface atoms of the ITO substrate [8,30]. At the other end, the coupling between the tip and the molecule (terminal) can enable electron tunneling and may alter the SAM properties in different manners such as lattice distortions, charge rearrangements, and the formation of electric dipole moments [31,32,33,34,35]. It also should be mentioned that the chemi-adsorbed bonding on the substrate may fluctuate and even migrate from one atom to another one due to various stimuli such as Joule heating and an external bias [36,37,38]. Previously, thermally activated spontaneous breakdown of the chemi-adsorbed bond has been observed on Joule heating, which could lead to migration of bonding sites of the substrate [14,36,37,38]. In the absence of a trapping-de-trapping phenomenon, the bond fluctuation is the main source of noise or resistance fluctuation in the molecular wire [4,7,14,37]. Additionally, there could be some fluctuations in electrostatic interactions between the tip and the molecular wire due to a rather high electric field by a bias voltage on the tip. Hence, electrical noises in a common molecular wire are generated by the fluctuations in bonds at the substrate and the electrostatic fluctuations at the tip-molecule junction. On the other hand, in molecular wires with rather strong dipole moments, dipole-induced fluctuations may contribute significantly to the noise [39,40,41]. In the schematic diagram, we show a vector diagram depicting a dipole moment fluctuation. The *μ*_I_ is the initial dipole moment of the molecular wire that changes to *μ*_R_ after the application of an external electric field. The δ*μ* is the corresponding fluctuation in the dipole moment producing additional noises as δ*μ* could affect the tunneling currents at the junction.

### 3.3. Resistance Estimation of the Individual Molecular Wire from the Current Image of SAMs

**Figure 2:**
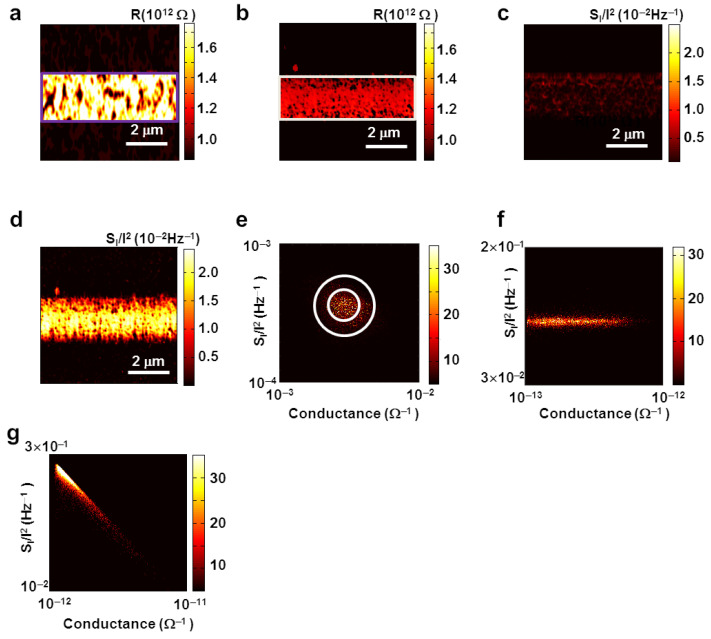
Resistance and noise mapping and their inter-dependence in HSAM and FSAM molecular wire patterns. (**a**) An individual molecular wire resistance map for a HSAM pattern (bright area). The dark area represents bare ITO surface. The order of resistance values on the HSAM regions was of ~TΩ. (**b**) An individual molecular wire resistance map for a FSAM pattern (bright regions) showing ~TΩ of resistance. (**c**) A noise *PSD* map for HSAM (bright regions). The order of the noise was 10^−2^ Hz^−1^. (**d**) A noise *PSD* map for FSAM (bright regions) showing a rather large noise with an order of 10^−1^ Hz^−1^. (**e**) A double histogram plot for noise *PSD* versus conductance on a log-log scale for the ITO. The concentric circular data indicate the typical behavior of a metallic point contact at the junction of the tip and ITO surface. (**f**) A double histogram plot for noise *PSD* versus conductance on a log-log scale for HSAM. The plot shows that noise *PSD* was nearly independent of the conductance. This is typical behavior for alkane-chain-based non-polar molecular junctions. (**g**) A double histogram plot for noise *PSD* versus conductance on a log-log scale for FSAM. Noise *PSD* scaled as ~*G*^−2^ implies a tunneling barrier fluctuation.

Figure 2a,b show the resistance maps of HSAM and FSAM patterns on ITO substrates, respectively. The map of averaged resistance (*R*) values for individual molecular wires in the SAM patterns was obtained from the corresponding current maps measured at 0.1 V. The current (*I*) measured through the c-AFM probe is proportional to the contact area (*A*) between the tip and SAM, and the packing density of the molecular wires (*n*) in the SAM. Hence, the R of each wire in the SAM can be estimated as *R* = *A*(*nV*/*I*), assuming the wires are parallel to each other [14]. By substituting the variables with typical values for the contact area between our tip and the SAM (close to the actual area of the probe ~2000 nm^2^) and the packing density of a patterned SAM on the ITO substrate (~2 nm^−2^) [12,13,14,15], we could obtain the resistance map of an individual molecular wire. The averaged resistance map of the HSAM pattern on the ITO substrate showed an individual molecule resistance value of ~1.6 × 10^12^ Ω in the 2 μm width, which was consistent with the previously measured resistances in alkane chain molecular wires [14,15,16]. Previously, lateral force microscopy imaging has been extensively utilized to show the formation of thin SAM patterns because it is often difficult to obtain a clear contrast on topography images of such a thin molecular layer on a rather rough surface [42,43]. In our case, the lateral force images of our SAM patterns exhibited a clear contrast, confirming the formation of the SAM pattern (Supplementary Materials, Figure S1). The resistance map for the FSAM molecules shows an individual molecule resistance value of ~1.2 × 10^12^ Ω, which is comparable to that of the HSAM molecular pattern, despite the fact that the length of the FSAM molecules was much shorter than that of the HSAM [16,24]. As a control experiment to analyze the substrate roughness, we also imaged a bare ITO and other substrates, showing that our ITO substrate had a flat surface comparable to that of other commonly-used substrates such as bare Au (Figures S1 and S2 in Supplementary Materials). The root-mean square (RMS) values of surface roughness for the *ITO* and the *gold* surfaces were *6* and *4* nm, respectively. It supports the notion that the resistance maps originated from molecular layer patterns on the ITO substrate. The rather large resistance in the FSAM can be explained by the fluorination of the molecules. Note that the resistance of a molecular wire of the same species increases exponentially with the length (*d*) of a molecular wire, following the relationship as in [14,15,16]

(1) $$R\;=\;R_{0}\exp(\alpha d)\;$$

where α is a tunneling attenuation factor that depends on the barrier height, and pre-factor *R*_0_ depends on junction resistances [13,14,15,16]. Previous works showed that, due to fluorine atoms, the electron cloud is skewed towards the terminal (fluorine atoms), resulting in a rather large contact resistance due to the poor coupling of the molecule with substrate and the obstruction in the charge transmission [16,24]. These effects could lead to much larger *R*_0_ and α for FSAMs than HSAMs, increasing the overall resistance of a fluorinated molecule by two or three orders of magnitudes [16,24]. For example, the resistance values of similar alkane molecules (C-8) with or without fluorine groups can differ by three orders of magnitude [15]. We chose a much smaller length of the FSAM molecule so that the resistance and noise would be comparable to those of the HSAM. Our results show that the substitution of atoms in a molecular wire can indeed significantly change the transport properties of a SAM.

### 3.4. Correlation between Conductance and Noise in HSAMs and FSAMs

Figure 2c,d are the maps of current normalized noise *PSD* (*S*_I_/*I*^2^) spectra measured on the HSAM and FSAM, respectively. Here, we mapped the noise values (*S*_I_) at a central frequency of 17.2 Hz along with the topography and current maps via the scanning noise microscopy. A current-normalized noise *PSD* (*S*_I_/*I*^2^) map was obtained by dividing the *S*_I_ values in the *S*_I_ map by the corresponding current values in the current map. The *PSD* of the HSAM was of the order of 5 × 10^−3^ Hz^−1^, whereas the FSAM exhibited an order higher *PSD* of ~1 × 10^−2^ Hz^−1^. Previous works showed that longer molecular wires usually exhibit larger noises as well as larger resistance values than shorter ones [40]. However, in our case, an HSAM pattern with longer molecular length and higher *R* had a lower noise level than FSAM patterns. Presumably, a stronger dipole in a C–F bond could create defects and disorder in the FSAM, resulting in a higher noise level, as reported previously [23,24]. These results show that the noise *PSD* is very sensitive to molecular species in a SAM, and it can be utilized as a means to distinguish between different molecular wires.

Figure 2e,f are two-dimensional histogram plots showing the relationship between the noise *PSDS*_I_/*I*^2^ and the conductance on a log-log scale for bare ITO and molecular wire (HASM) coated ITO surface, respectively. The graphs were obtained by statistical weightage plotting of the conductance and noise *PSD* data on *x* and *y* axes, respectively. For the ITO, noise *PSD* amplitude *S*_I_/*I*^2^ showed some variations in terms of conductance change over the ITO surface. The variation in the noise *PSD* was mostly confined within two concentric circles, as marked with white lines in Figure 2e. The noise *PSD* and conductance variations on the surface of the ITO were small, implicating an electrically homogeneous surface [44]. Such a distribution centered in the small region of conductance and noise *PSD* could be attributed to the mechanical oscillations of the direct point contact between the tip and the surface atoms, as reported previously [44,45]. The data in the inner circle represent the mechanical oscillations of the atoms in direct contact with the tip, whereas the outer circle regions indicate those from neighboring atoms [44,45]. This implies that the oscillations of neighboring atoms could affect the conductance and the noise severely, and hence we can expect different conductance and noise levels depending on the materials of the tip and the surface, where the coordination numbers and packing arrangements of atoms vary from material to material. In this case, there was a direct coupling between the atoms of the ITO and the tip. Conversely, there was no direct contact between atoms of the surface and the tip in the case of HSAM, and they were connected through molecular wire (Figure 2f). Here, the noise *PSD* of HSAM (*S*_I_/*I*^2^) shows no significant dependence on the conductance, and noise *PSD* varies parallel to the conductance axis (*x*-axis), which is consistent with the results for typical alkane-based SAMs, as reported previously [12,46]. One can derive the noise *PSD* *S*_I_/*I*^2^ from the resistance fluctuation, as reported previously [14].

By differentiating the Equation (1) for a molecular wire resistance, we obtain:

*S*_I_/*I*^2^ = <δ*R*^2^>/*R*^2^ = <δ*R*_0_^2^/*R*_0_^2^> + *d*^2^ <δ*α*^2^> + *α*^2^ <δ*d*^2^>. (2)

Here, the first term comes from the fluctuation of contact resistance between the substrate and molecules [14]. The second term represents the tunneling barrier fluctuations [4]. Here, *α* is related to the tunneling barrier height like *α* = (2*m*_e_*Φ*)^1/2^/*ħ*, where *m*_e_ and *Φ* are an *effective electron mass* and a *tunnel barrier height*, respectively. The third term originates from the fluctuations of the molecular length *d* due to torsion and chain twisting [14]. Previous studies showed that in the case of alkane chain-based molecular wire layers (such as HSAM) at a low bias condition, there was not much fluctuation in the barrier height and the molecular length, and the contact resistance fluctuation (first term in Equation (2)) caused by bond fluctuations dominated its electrical noises [4,14,46]. In this case, we can consider only the first term of Equation (2), while ignoring other terms. Note that the molecule-substrate contact resistance *R*_0_ is determined by the binding of molecules on the substrate and is expected to be the same for any molecules that bind to the substrate with identical chemisorbing groups. Previous works showed that different molecular species, as long as they bound to the substrate with the same chemisorbing groups, exhibited a constant value of <δ*R*_0_^2^/*R*_0_^2^> [14,44,45,46]. Since all HSAM molecules in the SAM bound to the substrate using the same silane groups, we can expect that <δ*R*_0_^2^/*R*_0_^2^> should remain constant and independent of the location on the SAM. Thus, from Equation (2), we can expect almost a constant value for noise *PSD* *S*_I_/*I*^2^, which is consistent with our results (Figure 2f) [14,44,45,46].

Figure 2g is a two-dimensional histogram plot showing the relationship between noise *PSDS*_I_/*I*^2^ and conductance on a log-log scale for the FSAM. The noise *PSD* in the FSAM showed a strong negative correlation with its conductance. In this case, the noise *PSD* *S*_I_/*I*^2^ decreased with the increase in the conductance *G*, following the relationship like *S*_I_/*I*^2^ ∝ *G*^−2^. Previously, such scaling behavior of the noise *PSD* with conductance has been explained by different electrical characteristics of the SAM [2,4,44,45,46,47]. For example, the scaling behavior and current noises in non-polar molecular wires were attributed to the characteristics of the tunneling currents and resistance fluctuations in molecular wires [44,45,46,47]. However, in the FSAM, the formation of strong dipoles could lead to a significant fluctuation in the tunneling barrier at molecular junctions [24]. This tunnel barrier fluctuation could lead to the fluctuation in the attenuation factor *α*. The noise due to the dipole fluctuation is relatively high because of the direct coupling with the tip, and it can exceed the noise due to the contact resistance fluctuation [48]. Previous works showed that the induced dipole in molecules could cause the dipole fluctuation with its relaxation time in the range of milli-seconds [38,39,45,46,47]. Such dipole fluctuations in individual molecules could work as a noise source to generate electrical current noises, as we observed (Figure S3, Supplementary Materials) [14]. However, it should be mentioned that the generation process of noises, in general, is a random process, and thus the dipoles of all molecules in our sample do not fluctuate simultaneously. When a dipole-induced noise is a dominant term, the noise *PSD* can be written as *S*_I_/*I*^2^ ≅ *S*_I-dipole_/*I*_dipole_^2^, where *I*_dipole_ is the current including the effect of electric field redistribution induced by the dipole in the molecule, and *S*_I-dipole_ is the corresponding fluctuation in the current. Previous works showed that the dipole-induced noise *S*_I-dipole_ is proportional to the square of the dipole moment fluctuation Δ*μ* like *S*_I-dipole_ ~ (Δ*μ*)^2^ [48,49,50]. Additionally, the charge transfer rate is reported to be proportional to the square of the dipole moment, and thus *I*_dipole_ should be proportional to the second power of the dipole moment like *I*_dipole_ ~*μ*^2^ [49,50]. At a rather small fixed bias condition, we can assume that Δ*μ* is much smaller than *μ*, and dipole fluctuations Δ*μ*, as well as *S*_I-dipole_, are not affected by the variation of *I*_dipole_, which is also shown in our experimental data (Figure S4, Supplementary Materials). In this case, since *I*_dipole_ is proportional to the conductance *G*, we can obtain *S*_I-dipole_/*I*_dipole_^2^ ∝ 1/*G*^2^, as we observed. Our results implicate that the scaling behavior of noise *PSD* with conductance has a different origin in each of the different species of molecules, showing the versatility of our analysis method.

### 3.5. Evidence of Dipolar Interaction in FSAMs

**Figure 3:**
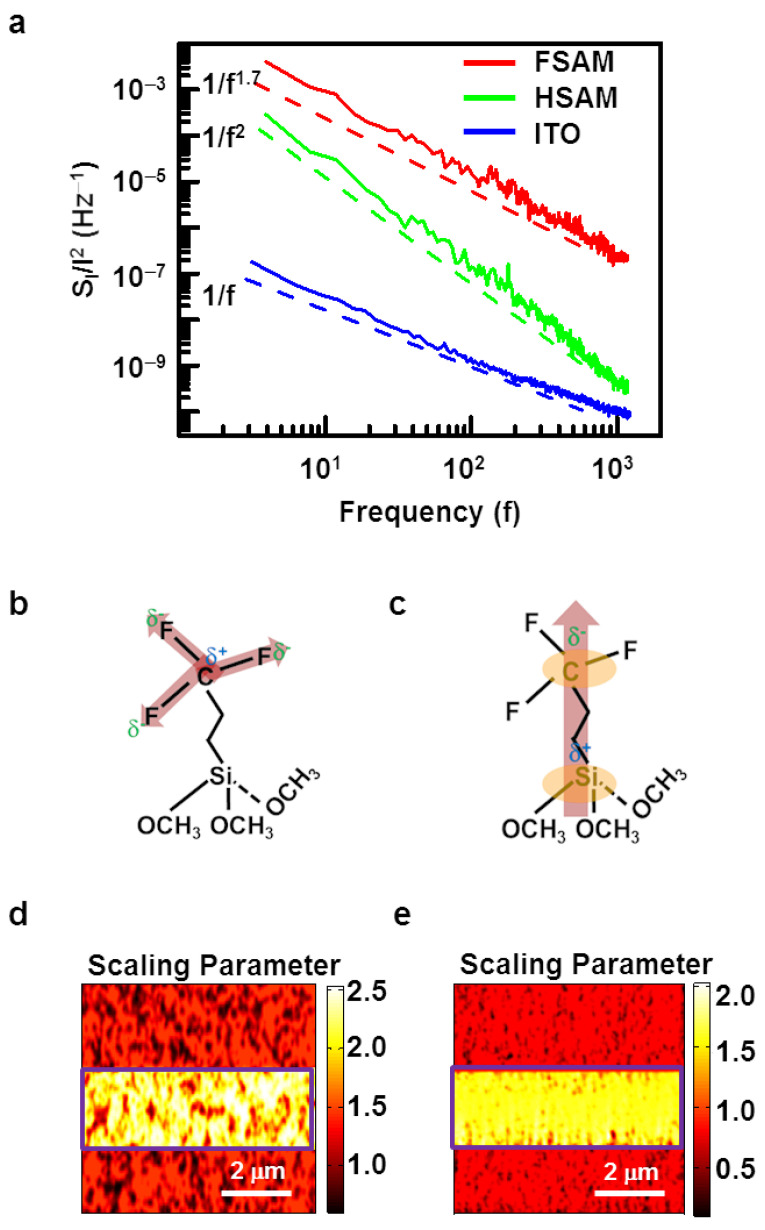
The frequency dependence of noise *PSD* and its scaling behavior in HSAM and FSAM. (**a**) Noise *PSD* as a function of frequencies in HSAM, FSAM, and ITO. HSAM exhibited 1/*f*^2^ behavior supposedly due to bond fluctuations. FSAM showed a weaker dependence like *PSD* ~1/*f*^1.7^, which can be attributed to the effect of dipole fluctuations. The ITO shows typical 1/*f* behavior commonly observed on a bulk film. (**b**) A schematic diagram of the bond polarization of FSAM molecule. Due to high electronegativity of fluorine, bonds are strongly polarized. (**c**) A schematic diagram showing backbone polarization of the FSAM. (**d**) A scaling parameter map of noise *PSD* spectra measured on HSAM (bright regions). HSAM showed a scaling parameter of 2. (**e**) A scaling parameter map of noise *PSD* spectra measured on FSAM (bright regions). FSAM regions showed scaling parameter of 1.7.

Figure 3a shows the frequency dependence curves of normalized noise *PSD* (*S*_I_/*I*^2^) in the FSAM, HSAM, and ITO substrates plotted with red, green, and blue colors, respectively. The dotted lines are placed to guide the eye. The spectra were measured using a fast-Fourier transform (FFT) spectrum analyzer (SRS SR770) while keeping the conducting AFM probe stationary at a fixed location on the SAM or the ITO substrate. The overall noise *PSD* of SAMs was larger than ITO substrates, indicating the increased current noises at the junctions with molecular wires. Presumably, the fluctuation of bonds at molecule-ITO junctions as well as dipole moments could contribute to the increased electrical noises in SAMs. Interestingly, SAMs and ITO substrates exhibited quite different scaling behaviors. The data of the ITO substrate can be fitted by 1/*f*, which is typical for the system with multiple noise sources with different trapping times *τ* [7,51]. Previous works showed similar scaling behavior for bare Au and other conducting substrates without molecular layers [14]. On the other hand, the data on the HSAM layer can be fitted by 1/*f^2^*, which is also typical behavior for well-ordered SAM without many defects, as reported previously [14,47,51]. Previous works showed that in the junctions of molecular wires with a rather weak dipole moment such as HSAM, the bond fluctuations between molecules and substrate generate electrical noises, which can be modeled via the conductance fluctuations in two-level conducting states having the constant characteristic relaxation time *τ* [46,51,52]. In this case, the relaxation function φ(t) for a specific decay process can be assumed to obey a Debye relaxation, and in the time domain, the relaxation function can be expressed as *φ*(*t*) ~*exp*[−*t*/*τ*] [53,54,55,56]. As a result, noise *PSD* has a Lorentzian form like *PSD* ∝ *τ*/[1 + (2π*fτ*)^2^], and, at sufficiently high frequencies, it can be approximated as ~1/*f*^2^, which is consistent with the data of the HSAM [14,53]. However, the data of FSAM can be fitted by ~1/*f^β^* with a rather different exponent *β* of ~1.7. One plausible explanation can be that the strong dipole of the FSAM could cause the fluctuation in the tunnel barrier and eventually slow down the decay process in the two-level conducting states of the noise model [50,52]. In such a case, the distribution function gets stretched on a time scale and can be modeled using the Kohlrausch–Williams–Watts (KWW) relaxation function as in [56,57].

*φ*(*t*) ~*exp*[−*t*/*τ*]^1−γ^ (3)

In principle, 1−γ could take a value between 0 and 1. In the presence of such a distribution function, the noise *PSD* takes the form of *S*(*f*) ~1/*f*^2−γ^ [53,56,57]. For strongly disordered, heterogeneous, and interacting dielectric systems, the decay processes are very slow and γ could be as high as 0.6 [57]. In the case of FSAM, γ can be estimated as ~0.3, indicating a significant fluctuation of tunneling barriers, presumably due to the strong dipole moments of the FSAM. These results are in accordance with our assumption that the attenuation factor *α* (Equation (2)) is significantly affected in a FSAM.

Figure 3b,c are schematic diagrams showing the formation of dipoles in the terminal and the backbone chain of the FSAM molecular wire, respectively. Despite its rather small molecular length, the FSAM showed higher resistance and electrical noises than those of the HSAM. A plausible explanation for this is the presence of fluorine atoms on its terminal group. A fluorine atom has very high electronegativity and, thus, can form strong dipoles in an atomic bond with carbon (Figure 3b). The strength of the C–F dipole is 1.4 Debye, and that of the C–H is 0.3 Debye [20]. The large difference in the dipolar strength could have strong effects on a densely packed FSAM. A single molecular wire has three C–F dipoles, and they can interact with the intra and inter-molecular dipoles of the molecular wires. These could lead to strong dipolar interactions in the molecular wires of the FSAM, creating more substantial barrier fluctuations and larger noises than the HSAM. Additionally, the possible formation of dipoles in the backbone chain of the molecular wire (see Figure 3c) could also contribute to the noises.

Figure 3d,e show the scaling parameter map on the HSAM and FSAM, respectively. The maps of the scaling parameter of the SAMs are differentiated by rectangular boxes from the ITO part. Here, the noise *PSD* values were measured in the frequency range of 17.3−1732 Hz at each point of the SAM surface and utilized to estimate the scaling factors. We fitted the *PSD* spectra with a function of *A*/*f^β^* to obtain the scaling parameter *β*. The *PSD* spectra on the molecular monolayer exhibited the scaling behavior. The HSAM exhibited a scaling factor (*β*) close to 2, whereas the FSAM exhibited a scaling factor of 1.7. Note that the scaling factor map shows a clear contrast between the SAM regions and the ITO regions (*β* ~ 1.0). We could consistently obtain the scaling parameter from different molecular wire samples in this study, supporting the notion that measured noise signals in a molecular monolayer were from the molecular parts rather than other conducting parts in the sample. These results show that the scaling parameter analysis could be utilized to discriminate between molecular species present on a nanoscale junction.

### 3.6. Effect of High Biases on the Transport in HSAMs and FSAMs

**Figure 4:**
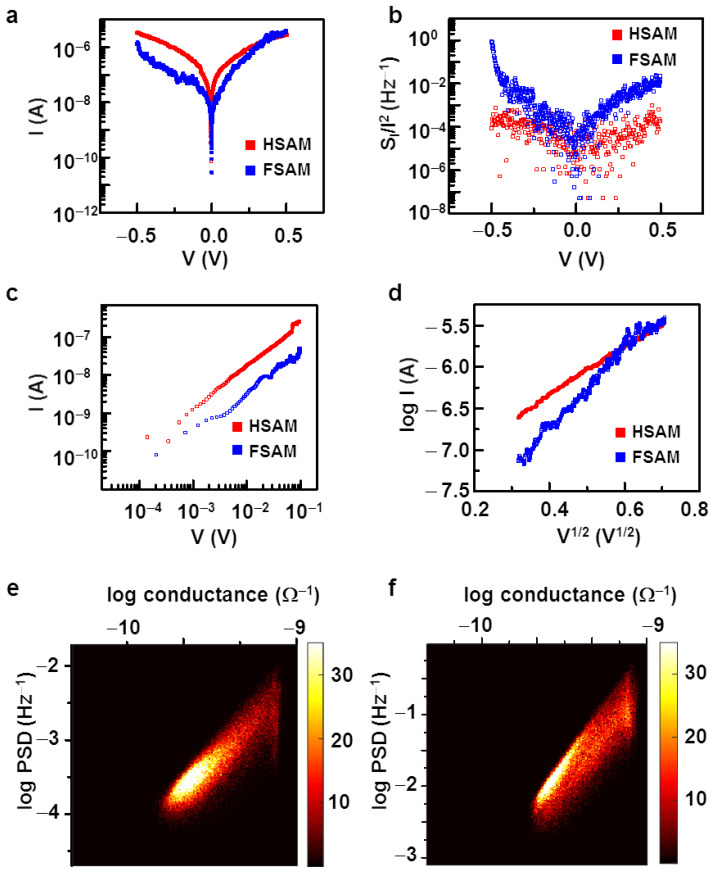
The effect of high biases on the conduction mechanism and noises in molecular wire junctions. (**a**) A scatter plot showing I–V curves of HSAM and FSAM on a semi-log scale. The curve was nearly symmetric for HSAM, whereas FSAM showed asymmetry and higher currents in positive bias cases. (**b**) Noise *PSD* dependence on the voltage for HSAM and FSAM. The noise *PSD* shows an asymmetric behavior in FSAM. (**c**) The I–V curves of HSAM and FSAM for low biases (below 0.1 V) on a log-log scale, showing the linear dependence of the current on voltage. (**d**) *log*I–V^1/2^ curves of HSAM and FSAM for high biases (above 0.1 V). The linear dependence of the curves implicates thermionic emission of carriers at high biases. (**e**) A double histogram plot for noise *PSD* versus conductance for HSAM at a high bias, showing a positive correlation. (**f**) A double histogram plot for noise *PSD* versus conductance for FSAM at a high bias. The positive correlations in high bias conditions implicate a change in the conduction mechanism from tunneling to thermionic emission.

A high electric field in one-dimensional molecular wires can significantly affect their transport and noise characteristics. Figure 4a shows typical current-voltage (I–V) curves on a semi-log scale for the HSAM and FSAM plotted with red and blue colors, respectively. For the measurement, a bias voltage on the ITO electrode was ramped from −0.5 to 0.5 V while keeping the Pt tip as a zero bias. The HSAM showed a nearly symmetric I–V curve. On the other hand, the FSAM showed an asymmetric behavior, where the current was higher by an order in the positive ITO electrode bias cases than that of the negative bias conditions. For example, in the case of 0.5 V or −0.5 V bias on the electrode, the rectification (*I*(0.5 V)/*I*(−0.5 V)) was ~3.5. Presumably, the presence of fluorine atoms induced the formation of dipoles at terminal groups and backbone on the FSAM, resulting in the asymmetric I–V curve [58,59,60]. One explanation for the asymmetric I–V curve at a rather large bias is the rearrangement of molecular dipoles by a large external bias voltage [61]. Previous works showed that, when molecules in SAM have a permanent dipole moment, a rather large external bias from an AFM probe can affect the dipole moments and change the thickness of the SAM, resulting in a conductance change of the SAM [62]. In our case, the fluorine groups at the end of the molecules in the FSAM are known to have negative charges. When a positive bias was applied to the conducting AFM probe, the fluorine groups were attracted toward the probe (i.e., negative bias to the ITO substrate), making the FSAM thicker [20,62]. We observed three times larger current levels at a rather large positive bias than at a negative bias, while the current levels were found to be rather symmetric at a small bias. This indicates that the induced dipole moment as a large negative bias made the SAM layer thicker and decreased conductance, which is consistent with previous reports [61,62,63,64,65].

Figure 4b shows the noise *PSD* dependence on voltage for the HSAM and FSAM, plotted with blue and red colors, respectively. The noise *PSD* was measured at 17.32 Hz along with the currents, while the voltage was swept from −0.5 to 0.5 V on the ITO surface. We observed symmetric noise *PSD* in the HSAM, while the noise *PSD* in the FSAM was asymmetric, with high values at negative biases. The increased noise in the FSAM at a negative bias on the ITO substrate could be associated with the stronger effective dipole formation at an applied negative bias. Previous studies showed that a stronger dipole could lead to a more substantial fluctuation in the tunneling width and tunneling barrier, resulting in high noise levels, which is also consistent with our results [61,62,63,64,65].

Figure 4c shows the *logI* versus *logV* curves for the HSAM and FSAM at a rather low bias below 0.1 V. Note that both data can be fitted by a curve with a slope of ~1, indicating a linear dependence of a current on the bias voltage at a low bias regime. Previous works showed that at small biases, electrical currents in a molecular junction can be explained by a tunneling current with a linear dependence on *V* like  I∝Vexp[−αd], which is consistent with our results [66].

On the other hand, the electrical currents at a rather high bias exhibited quite different behavior. Figure 4d shows the *logI* versus *V*^1/2^ curves for the HSAM and FSAM at high biases (above 0.1 V) plotted with blue and red colors, respectively. In both the curves, *logI* showed a linear dependence on *V*^1/2^, which is consistent with previously reported results of thermionic conduction in molecular wires at a large bias voltage [66,67]. It is worth mentioning that other transport mechanisms such as tunneling and field emission can have a similar voltage dependence behavior. However, previous works showed a temperature dependence of molecular conduction, strongly supporting thermionic emission rather than other mechanisms such as tunneling or field emission, which cannot have a temperature dependence [66,67].

Figure 4e,f are the two-dimensional histogram plots showing the relationship between noise *PSDS*_I_/*I*^2^ and conductance *G* at 0.5 V on a log-log scale for the HSAM and FSAM, respectively. The graphs were obtained by statistical weightage plotting of the conductance and noise *PSD* data at 0.5 V on the *x* and *y* axes, respectively. The noise *PSD* showed a strong and positive correlation with conductance in both HSAM and FSAM, following scaling behavior of *S*_I_/*I*^2^ ∝ *G*^2^. This dependence is opposite to the low bias scaling behavior of *PSD* on conductance, where we observed a negative correlation like *S*_I_/*I*^2^ ∝ *G^−^*^2^ (see Equation (2)). The positive correlation could be explained by electrical noises in the thermionic emission at a high bias. A previous report showed that, in the thermionic emission, the carriers diffuse due to thermal energy by Joule heating, and the noise due to thermal diffusion of carriers is proportional to the square of a change in temperature like *S*_I_/*I*^2^ ∝ (Δ*T*)^2^ [68]. At a molecular junction with its conductance *G*, the temperature change Δ*T* by Joule heating should be proportional to the power generated by electrical currents such as Δ*T* ∝ *GV*^2^. Since the bias voltage *V* was kept constant during the scanning, the noise *PSD* can be written as *S*_I_/*I*^2^ ∝ *G*^2^ at a high bias condition where the thermionic noise dominates. These results show that, at a rather high bias, a thermionic fluctuation can be a dominant noise source at molecular wire junctions.

## 4. Conclusions

In summary, we report a strategy to directly map the effects of dipolar interactions on electrical transport and noise in the self-assembled monolayers (SAMs) of molecular wires. In this strategy, the current and noise images were measured simultaneously on molecular wire patterns by using a conducting probe. Then, the maps were analyzed to extract characteristic parameters of dipolar noises. The FSAM molecular junctions exhibited the molecular resistance comparable with the HSAM of nearly six times longer in length, which could be due to the strong dipole formations in the FSAM obstructing the charge conduction. Furthermore, in the FSAM, the normalized noise-*PSD* scaled as the inverse of the square of the conductance like *PSD* ∝ *G*^−2^, which was explained by stronger dipolar interactions affecting the tunneling barrier in the FSAM. In addition, the frequency dependence of noise *PSD* showed 1/*f*^2^ behavior in the HSAM, whereas the FSAM showed a deviation, exhibiting 1/*f*^1.7^ dependence that could be attributed to the strong dipole formation slowing down the tunneling process. We observed asymmetrical current and noise only in the FSAM, owing to the strong dipole formation. Interestingly, at high biases, the conduction in HSAM and FSAM molecular wires showed the transition from tunneling to thermionic transports, which was reflected in the positive correlation of the noise *PSD* with conductance like *PSD* ∝ *G*^2^. Our results provide valuable insights about dipole noise generations in molecular wires with polarization, which could be helpful in understanding the charge transports and noise properties of molecular devices. Furthermore, we can expect that this strategy may be extended to the development of new methods for the analyses of other molecular wires with versatile non-linear behaviors such as memory effects and phonons.

## Data Availability

The data presented in this study are available on request from authors.

## Supplementary Materials

The following supporting information can be downloaded at: https://www.mdpi.com/article/10.3390/nano12081371/s1, Figure S1. Atomic force microscopy images on HSAM and FSAM prepared by a microcontact printing method on ITO substrate. (a) Topography image of HSAM patterns on ITO substrate. (b) Lateral force microscopy (LFM) image on the HSAM. We can clearly see the contrast of the lateral force values in the HSAM region and the bare ITO region. (c) AFM topography image of FSAM patterns. (d) LFM image measured on the FSAM pattern showing clear contrast between the FSAM pattern and ITO. The scale bars are 1 μm; Figure S2. Atomic force microscopy topography image of 50 nm thick Au film-surface on a SiO_2_ substrate. The scale bar is 500 nm; Figure S3. Noise *PSD* of FSAM molecules as a function of time; Figure S4. Dependence of noise (*S*) on current (*I*) on FSAM. The results show that *S* values exhibited a rather large fluctuations, while it does not change much with different *I* values.
