# Supplementary material for: Dipolar Noise in Fluorinated Molecular Wires
Source: Nanomaterials (Basel). 2022 Apr 16;12(8):1371. doi: 10.3390/nano12081371 (PMC9031467; doi:10.3390/nano12081371)
Supplement: Supplementary file 1 [file nanomaterials-12-01371-s001.zip › nanomaterials-1637755-supplementary-done.pdf]

nanomaterials

MDPI

Supplementary Material

Dipolar Noise in Fluorinated Molecular Wires

Mingyu Jung †, Shashank Shekhar *,†, Duckhyung Cho, Myungjae Yang, Jeehye Park and Seunghun Hong *

Department of Physics and Astronomy, Seoul National University, Seoul 08826, Korea; tririver@snu.ac.kr (M.J.); choduckhyung@gmail.com (D.C.); goodymj@snu.ac.kr (M.Y.); jhpark6521@snu.ac.kr (J.P.)

* Correspondence: shashank.iisc@gmail.com (S.S.); seunghun@snu.ac.kr (S.H.)

† These authors contributed equally to this work.

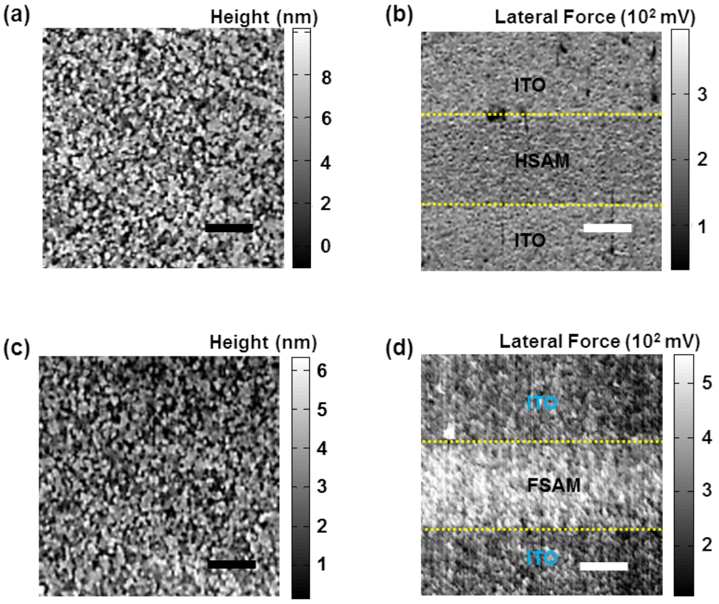

Figure S1. Atomic force microscopy images on HSAM and FSAM prepared by a microcontact printing method on ITO substrate. (**a**) Topography image of HSAM patterns on ITO substrate. (**b**) Lateral force microscopy (LFM) image on the HSAM. We can clearly see the contrast of the lateral force values in the HSAM region and the bare ITO region. (**c**) AFM topography image of FSAM patterns. (**d**) LFM image measured on the FSAM pattern showing clear contrast between the FSAM pattern and ITO. The scale bars are 1μm.

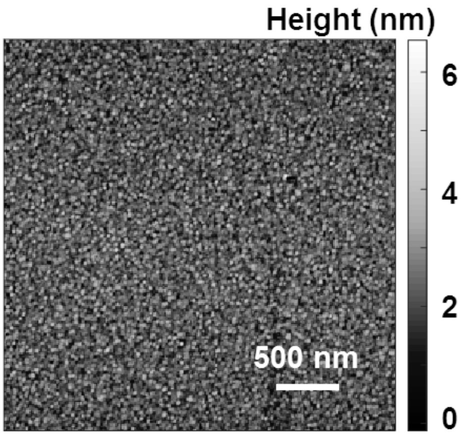

Figure S2. Atomic force microscopy topography image of 50 nm thick Au film-surface on a SiO_2 substrate. The scale bar is 500 nm.

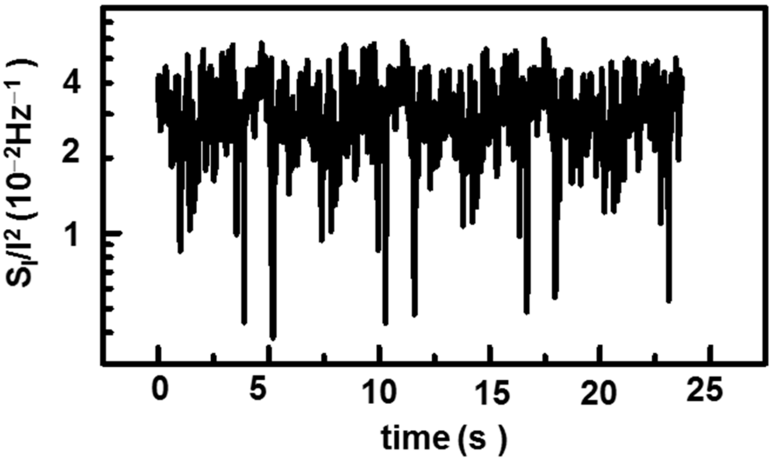

Figure S3. Noise PSD of FSAM molecules as a function of time.

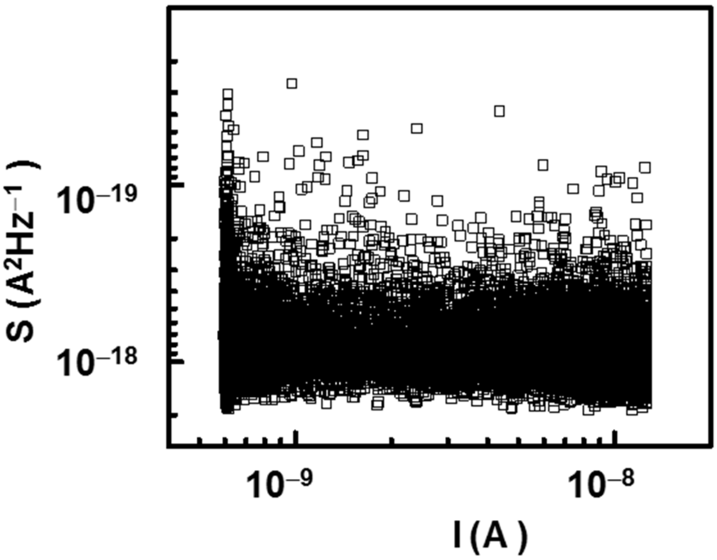

Figure S4. Dependence of noise (*S*) on current (I) on FSAM. The results show that *S* values exhibited a rather large fluctuations, while it does not change much with different *I* values.
